# Polygenic risk modulates myocardial repolarization and T-wave geometry in congenital long-QT syndrome type 1: evidence from digital ECG phenotyping

**DOI:** 10.3389/fcvm.2026.1736409

**Published:** 2026-04-23

**Authors:** Elinor Tzvi-Minker, Sven Dittmann, Christian Krijger Juárez, Andreas Keck, Eric Schulze-Bahr

**Affiliations:** 1 Syte Institute, Hamburg, Germany; 2 Department of Cardiovascular Medicine, Institute for Genetics of Heart Diseases (IfGH), University Hospital Münster, Münster, Germany; 3 Department of Experimental Cardiology, Amsterdam University Medical Center, Amsterdam, Netherlands

**Keywords:** electrocardiogram (ECG), long-QT syndrome (LQTS), polygenic risk score, QT interval, T-wave morphology

## Abstract

**Introduction:**

Congenital long-QT syndrome type 1 (LQT1), one of the major LQTS subtypes caused by pathogenic variants in the KCNQ1 gene, exhibits marked phenotypic variability, including incomplete penetrance and differences in myocardial repolarization. This variability suggests that additional genetic factors, particularly the additive effects of common single nucleotide polymorphisms (SNPs) captured by polygenic risk scores (PRS), may modulate disease expression.

**Methods:**

We analyzed digital 12-lead ECGs from 273 LQT1 patients carrying pathogenic KCNQ1 variants from a German national registry. Sixty quantitative descriptors of myocardial repolarization were derived using a validated, automated ECG-analysis algorithm. A PRS was calculated from 169 genome-wide SNPs recently known to affect QT interval duration in the general population. Associations between PRS and ECG parameters were tested using multivariable linear regression, including interaction terms and heart-rate correction of the QT interval and subsegments.

**Results:**

Higher PRS values were modestly but significantly associated with longer Bazett-corrected QTc intervals (β = 0.24, p = 0.00008) and longer T-peak to T-end (TPE) intervals (β = 0.16, p = 0.008). A novel finding was a significant negative interaction between T-wave area and T-wave duration, both measured and confirmed using Rautaharju-corrected parameters (β = −0.14, p = 0.006), suggesting that the polygenic burden modulates both timing and geometry of ventricular repolarization. The overall model explained a modest proportion of variance (R² = 0.09).

**Conclusions:**

Our findings demonstrate that a polygenic background modestly modulates not only QTc duration but also T-wave parameters in LQT1 patients, revealing rate-independent influences on repolarization dynamics. Despite the limited variance explained, these associations were statistically robust and trait-specific, suggesting biologically meaningful modulation of repolarization phenotypes. Quantitative ECG phenotyping combined with PRS may offer a scalable framework for dissecting genotype-ECG phenotype relationships in inherited arrhythmia syndromes, particularly when digital ECG data are used.

## 1 Introduction

Congenital long-QT syndrome (LQTS) is a potentially life-threatening inherited cardiac channelopathy that predisposes affected individuals to malignant ventricular arrhythmias and sudden cardiac death through delayed myocardial repolarization, reflected by QTc (heart-rate corrected QT) interval prolongation on the electrocardiogram (1). Among its several genetic subtypes, type 1 (LQT1) is the most common and results primarily from pathogenic loss-of-function variants in the *KCNQ1* gene that reduce the slow delayed rectifier potassium current Iks (2, 3). Although 75%–80% of LQTS patients have an identifiable, monogenic basis, considerable phenotypic heterogeneity exists even among individuals carrying identical pathogenic variants, indicating that common genetic variation and other, non-genetic modifiers contribute to disease expressivity (4, 5).

The QTc interval duration and T-wave morphology have been recognized as an important marker of myocardial repolarization and of arrhythmogenic potential (6, 7). Distinctive T-wave patterns, such as broad-based, biphasic, or low-amplitude configurations, are associated with specific LQTS genotypes and may provide prognostic information regarding arrhythmia triggers and arrhythmic risk (8–11). Quantitative ECG descriptors such as T-wave duration, T-wave area, and the T-peak-to-T-end (TPE) interval reflect temporal and spatial dispersion of repolarization and capture subtle electrical abnormalities that may precede overt QTc prolongation (12, 13). However, the extent to which a polygenic background may modulate these more subtle morphological ECG features in LQTS is only partly understood.

Polygenic risk scores (PRS) offer a quantitative framework to assess the cumulative phenotypic effect of several hundreds to thousands of common variants (SNPs) identified through genome-wide association studies (GWAS). Several large-scale studies have demonstrated that higher PRS values significantly associated with a prolonged QT interval (PRS-QT) in the general population (14) as well as in congenital LQTS (5) and drug-induced LQTS (15, 16). In addition, other ECG parameters such as P-wave duration, JT interval or QRS duration, have also been linked to polygenic risk profiles, with more than 400 associated SNP loci identified to date. Estimated heritability for ECG traits in European populations is approx. 29.3% for the QT interval, 18.2% for the PR interval, and 15% for the QRS duration (17–19), underscoring that additional genetic and environmental contributors remain to be elucidated.

Recently, PRS have been associated also with a prolonged, but heart rate-corrected QT/QTc intervals (PRS-QTc) both in the general population (17, 19) and among patients with LQT1-3 (20). Notably, these polygenic influences persist even within monogenic conditions such as LQTS, suggesting that common variants modulate electrophysiologic expressivity and penetrance beyond the effect of a single pathogenic mutation (5, 21). Recent work has further mapped cis-regulatory mechanisms at QT-associated loci, refining understanding of the molecular basis of polygenic modulation (22).

In contrast to most ECG intervals, the QT interval—and its major component, the JT interval—is highly dependent on the heart rate and therefore requires correction using established formulas, each with distinct assumptions regarding normalization to a heart rate of 60 bpm. Most PRS studies to date have analyzed baseline (uncorrected) QT intervals (PRS-QT), with relatively few assessing the association between PRS and heart rate–corrected QTc values (PRS-QTc). Furthermore, QT interval measurements in these studies were often derived from manual annotation or relied on automated outputs from ECG machines, generally without the application of a standardized approach such as the tangent method, which has been shown to yield the most accurate and reproducible QT measurements (23).

Despite these advances, the interplay between polygenic background and quantitative ECG morphology has not been systematically evaluated. In this study, we leveraged digital ECG data and high-resolution repolarization metrics, applying a recently validated, automated algorithm for QT interval computation using the tangent's method as well as components of repolarization (24). We correlated these digital ECG features with selected SNPs used for PRS-QTc calculation in the general population (18). Moreover we used the PRS-QTc to examine the potential relationship between PRS-QTc and T-wave morphology parameters in an exclusive cohort of LQT1 patients.

By narrowing our focus to a genetically and pathophysiologically homogenous group, and integrating monogenic, polygenic and digital ECG features, we aim to clarify how a polygenic background interacts with T-wave features that reflect characteristic dynamics of cardiac repolarization at rest—a step toward individualized and automated risk prediction and directed patient management in inherited arrhythmia syndromes.

## 2 Methods

### 2.1 Study population

In this study, 273 genotyped patients with congenital LQTS were included that had a pathogenic or a likely pathogenic (ACMG—American College of Medical Genetics and Genomics class 4 or 5) heterozygous variant in the *KCNQ1* gene (LQT1 subtype). Genetic variant curation was conducted as per American College of Medical Genetics and Genomics and Association of Molecular Pathology guidelines (25). All included participants were of European ancestry, consistent with the ancestry of the discovery cohorts used to derive the QT-PRS. The study was conducted in accordance with the Declaration of Helsinki (as revised in 2013) and approved by the institutional ethics board of University Hospital Münster, Münster, Germany (2021-315-f-S). An informed consent was available and signed by all study participants.

### 2.2 ECG recordings

Resting 12-lead ECG recordings in supine position were collected during routine outpatient care at the Institute for Genetics of Heart Diseases (IfGH) of the University Hospital Münster, Germany. ECGs were recorded with a speed of 50 mm/s for 10 s with a 500 Hz sampling frequency using an ECG machine (GE CAM-HD Registrator, GE Cardiosoft V6.73, GE HealthCare). The data were stored using the MUSE™ data management system (Muse v9 Cardiology Information System, GE HealthCare) and subsequently exported in an anonymized form in XML format. XML files were further converted into CSV files using freely available Python script. Thereafter, a previously published and custom-made MATLAB-based algorithm (24) was used to extract and calculate the following ECG parameters (in ms):

- RR interval: time interval between adjacent R peaks, averaged across the 10s ECG segment
- QT interval: time interval between Q onset and T-wave end (T-end) as determined by the tangent method (10)
- TPE: T-peak to T-end interval
- TPE/QT: TPE to QT ratio
- R-peak/T-peak amplitude ratio, T-wave normalized to R-peak amplitude
- T-wave area: the area under the curve between T-start and T-end, normalized to R-peak amplitude, calculated using trapezoidal numerical integration
- T-wave duration: T-start to T-end interval
- JT intervals: J to T-start, J to T-peak, J to T-end
- T-start to T-peak duration

The measured QT intervals were corrected to the heart-rate (HR, in bpm = 60,000/RR) for calculation of the corrected QT interval (QTc) by using several formulas:

- The Bazett's formula (QTc (B) = QT/√RR) (26) most widely used
- The Fridericia's formula (QTc (F) = QT/RR^(1/3)) (27)
- The Framingham method (QTc (FR) = QT + 0.154 × [1 − RR]) (28)
- The Hodges formula (QTc (H) = QT + 1.75 × [HR − 60]) (29)
- The Rautaharju formula (QTc (R) = QT − *β* × [RR − 1], with *β* = 0.185 for men and *β* = 0.288 for women) (30) using a sex-specific linear regression model derived from large population datasets, and thereby achieving more accurate rate correction over a wide range of RR intervals.

Together, the five most common methods for QTc calculation were applied, allowing appropriate selection based on study design, heart rate range, and population characteristics. ECG datasets were excluded from analysis if data quality was low (assessed both manually and by the algorithm). ECG parameters were determined in three ECG channels: II, V5 and V2. First, channel II was assessed and if no value was produced, channel V5 was assessed. Finally, if no value was produced from channel II or channel V5, channel V2 was used to determine the QT interval.

### 2.3 Genotyping and polygenic risk score calculation

We performed genome-wide genotyping, quality control imputation analysis for all LQT1 cases on the Illumina HumanOmniExpress array, as previously described (5). All genetic variants were mapped to and reported using Genome Reference Consortium Human genome build 37 (hg37).

The polygenic risk score (PRS) was calculated following strict quality control and recommended protocols to ensure reliability and interpretability, consistent with established guidelines. Initially, genotype data underwent rigorous curation, with samples excluded for excessive missingness (>4%), high inbreeding coefficients (F > 0.15), or duplication. The curated datasets were converted from PLINK to VCF format and submitted to the Michigan Imputation Server for haplotype-based imputation using the 1,000 Genomes reference panel (https://imputationserver.sph.umich.edu/). Imputation enhances genotype completeness and harmonizes variant representation across individuals, ensuring robust downstream analyses. Post-imputation, variants were pruned for linkage disequilibrium (LD) with the PLINK filter (indep-pairwise 50, 5, 0.05). This process employs a sliding window of 50 SNPs, moves in 5-SNP increments, and removes SNP pairs with LD above 0.05, thereby retaining only independent variants and minimizing inflation of PRS due to non-independence. Only SNPs with imputation R^2^ > 0.5 were retained to ensure high-quality variant calls. This step has therefore led to inclusion of 169 of the original 176 independent SNPs, previously shown to robustly associate with QT, JT and QRS interval variation in the general population with beta-effects between +3.97 and −4.7 ms per allele on the uncorrected QT interval (31). The full list of SNPs is described in the Supplementary materials, Table S2. These SNPs were derived from population-based GWAS in European ancestry cohorts; ancestry matching was maintained to minimize bias, but external validation in multi-ancestry datasets remains warranted.

For each study subject, these SNPs were then extracted from the post-imputation dataset, and individual genotypes were weighted by their GWAS-derived effect sizes as previously published. The personal PRS was then computed in R by summing the products of each risk allele and its corresponding effect size across the selected SNPs. The resulting anonymized dataset is included in the Supplementary materials, Data_sheet2.

These methodologic steps align with recommendations for maximizing PRS validity, addressing genotype quality, LD control, variant selection, and score computation. Rigorous QC and adherence to established protocols are essential for minimizing confounding and maximizing the predictive value of PRS in quantitative genetics and translational research.

### 2.4 Analysis of PRS and ECG parameter association using multivariable linear regression model

To assess the joint contribution of PRS and ECG features in the LQT1 cohort, we applied elastic net regression for variable selection and model fitting. Prior to modeling, all predictors were standardized. Both age and sex were included as covariates in the model. Elastic net regression was implemented in MATLAB using the *lasso* function with 10-fold cross-validation to tune the regularization parameter. The mixing parameter *α* was set to 0.5, which balances the penalty between L1 (lasso) and L2 (ridge) regularization. Nonzero coefficients were identified as selected predictors. To obtain unbiased effect size estimates, the subset of predictors retained by elastic net was then fitted using ordinary least squares linear regression (*fitlm* function in MATLAB).

Multicollinearity among predictors can inflate variance in regression coefficient estimates, making inference and variable selection unstable. A common preprocessing approach is to remove collinear predictors based on correlation thresholds or variance inflation factors (VIF). However, such manual filtering may lead to loss of information, especially when it's unclear which of the predictors play an important role in the model. By contrast, the elastic net penalty combines the sparsity of the lasso (L1​) with the grouping effect of ridge regression (L2). Using this hybrid regularization leads to stabilized coefficient estimates in the presence of collinearity. To further refine the model, stepwise linear regression was applied (*step* function in MATLAB) starting from the reduced predictor set, ensuring parsimony and retention of only the most informative variables for interpretation.

### 2.5 Modeling strategy and consideration of nonlinear effects

Given the high dimensionality and strong collinearity among ECG-derived repolarization features, we adopted a data-driven regularized regression framework rather than manually specifying nonlinear or quadratic terms *a priori*. Elastic net regression was chosen because it balances variable selection and coefficient stabilization in the presence of correlated predictors and allows interaction terms to be retained only if supported by the data. In contrast to outcome-driven models that explicitly engineer quadratic or higher-order interaction terms to optimize arrhythmic risk prediction [for example in Jin et al. (32)], our objective was to identify ECG features and interactions that explain variance in polygenic burden while minimizing overfitting in a moderate-sized, phenotype-focused cohort. Accordingly, interaction effects were permitted to enter the model through regularized selection rather than being forced *a priori*, resulting in the identification of a robust interaction between HR-corrected T-wave area and T-wave duration. This approach prioritizes interpretability and generalizability for genotype–phenotype mapping rather than maximizing predictive performance for clinical outcomes.

## 3 Results

Overall, a group of 273 LQT1 patients, imbalanced across sex (see Table 1), demonstrated comparable baseline ECG features (see Table 2), with predictable significant differences between males and females in QT, showing prolongation in males over females. In males, the T-wave duration (*p* = 0.004), JT-start interval (*p* < 0.00001, also called ST-segment) and T-start to T-peak duration (*p* = 0.002) were significantly longer compared to female LQT1 patients. The corrected digital ECG parameters could be viewed in the Supplementary materials, Table S1.

**Table 1:** Demographic characteristics.

Characteristic	Female	Male
*N*	182	91
Age at rest ECG	35.0 ± 16.5	30.1 ± 20.5

**Table 2:** Digital ECG parameters (mean ± SD) in 273 LQT1 patients.

Parameter	Female LQT1 patients	Male LQT1 patients	Statistical significance between genders (Wilcoxon Rank Sum Test)
	(*N* = 182)	(*N* = 91)	
RR interval (ms)	970.1 ± 198.0	958.9 ± 228.8	*P* = 0.9
QT (ms)	441.8 ± 54.3	424.4 ± 59.0	***P*** **=** **0.016**
QTcB (ms)	437.0 ± 40.6	451.0 ± 34.0	***P*** **=** **0.0001**
QTcF (ms)	431.9 ± 41.0	447.4 ± 36.3	***P*** **=** **0.0001**
QTcFR (ms)	424.0 ± 58.4	441.8 ± 54.3	***P*** **=** **0.016**
QTcH (ms)	423.9 ± 58.5	441.7 ± 54.3	***P*** **=** **0.016**
QTcR (ms)	424.0 ± 58.4	441.8 ± 54.2	***P*** **=** **0.016**
JT-start (ms)			
ST segment	124.6 ± 66.4	85.2 ± 59.9	***P*** **<** **0.00001**
JT-peak (ms)	293.7 ± 45.4	281.0 ± 45.9	*P* = 0.047
JT-end (ms)	368.0 ± 52.2	353.8 ± 52.6	*P* = 0.07
TPE (ms)	70.1 ± 15.1	68.8 ± 14.3	*P* = 0.5
TPE/QT ratio	0.16 ± 0.03	0.16 ± 0.02	*P* = 0.1
T-wave duration (TWD) (ms)	243.5 ± 70.1	271.2 ± 79.9	***P*** **=** **0.004**
T-wave area (TWA) (ms)	14.7 ± 11.7	15.1 ± 12.3	*P* = 0.8
T-start to T-peak (ms)	173.4 ± 58.2	198.2 ± 65.0	***P*** **=** **0.002**
R/T amplitude	3.9 ± 2.9	4.0 ± 3.0	*P* = 0.7

QTcB, Bazett's formula; QTcF, Fridericia's formula; QTcFR, Framingham method; QTcH, Hodges formula; QTcR, Rautaharju formula; J, J-point (end QRS).

Bold values indicate statistically significant differences (*p* < 0.05).

### 3.1 Multivariable linear regression analysis

In the linear regression analysis, the polygenic score (PRS) was modeled as a function of 10 ECG parameters (TPE, QT, TPE/QT, R/T amplitude, T-wave area, and T-wave duration, JT-start, JT-peak, JT-end and T-start to T-peak duration) uncorrected. In addition, the five formulas above were used to correct all frequency-dependent parameters (i.e., R/T amplitude was not corrected for the heart-rate), which led to a total number of 55 ECG predictors. Interactions between model parameters were not pre-specified. Age and sex were included as covariates.

**Figure 1:**
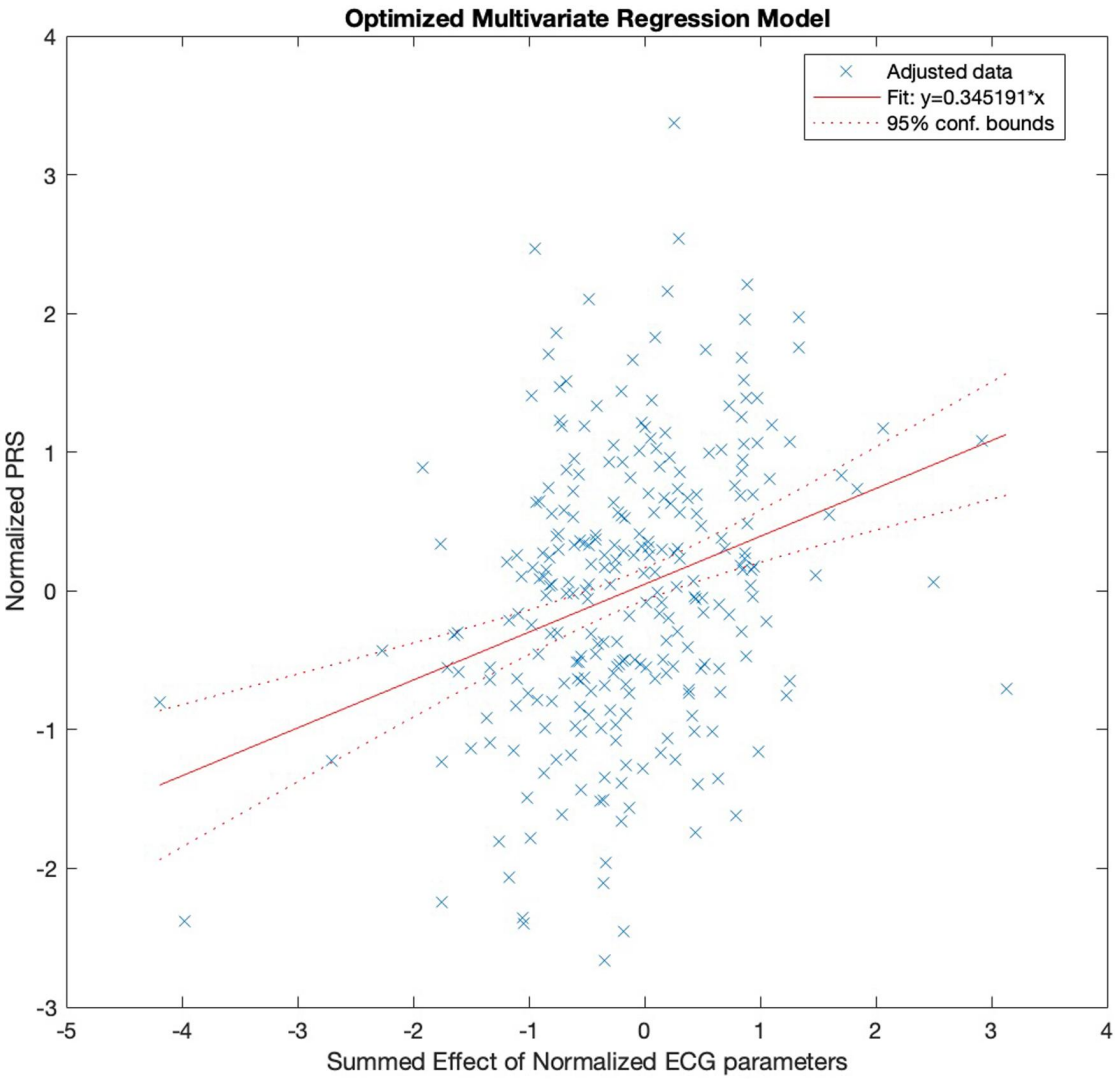
Reduced multivariate regression model between normalized PRS (*y*-axis) and summed effect of normalized ECG parameters in the model: PRS ∼ 1 + QTcB + TPEcR + TWAcR * TWDcR (Table 3) (*x*-axis). “x” represents individual data.

Following a 10-fold cross-validation Lasso regularization and step-wise refinement of parameters in the regression model, the overall explanatory power of the resulting model, as shown in Figure 1: PRS ∼ 1 + QTcB + TPEcR + TWAcR * TWDcR, was modest with R^2^ = 0.09, and model fit was highly significant (F-stat = 5.5, *p* = 0.00008).

Table 3 presents the model's parameters. We found that QTcB (QT corrected according to the Bazett formula) as well as TPEcR (TPE corrected according to the Rautaharju formula) emerged as main effects, demonstrating a positive association with PRS (QTcB: *β* = 0.24, *t*-Stat = 4.01, *p* = 0.00008; TPEcR: *β* = 0.16 *t*-Stat = 2.66, *p* = 0.008). This indicates that higher polygenic risk was associated with longer QTc, consistent with the notion that common genetic variation contributes to repolarization abnormalities in LQT1. The positive relationship between TPEcR and PRS indicates that polygenic burden not only prolongs the overall QTc but also enhances regional differences in repolarization, implying that common variants modulate both global and local electrophysiological processes.

**Table 3:** Estimated coefficients in the multivariable linear regression model.

Predictor	Estimate	SE	tStat	*p* Value
(Intercept)	0.05	0.06	0.78	0.43
**QTcB**	0.24	0.06	4.01	**0** **.** **00008**
TWAcR	−0.003	0.07	−0.05	0.96
TWDcR	−0.12	0.06	−1.90	0.06
**TPEcR**	0.16	0.06	2.66	**0**.**008**
**TWAcR×TWDcR**	−0.14	0.05	−2.71	**0** **.** **007**

QTcB, QT interval corrected according to Bazett; TPEcR, T-peak to T-end interval corrected according to Rautaharju; TWAcR, the area under the T-wave curve, corrected according to Rautaharju; TWDcR, the duration of the T-wave from T-start to T-end, corrected according to Rautaharju.

Bold values indicate statistically significant differences (*p* < 0.05).

Among the interaction terms we found that normalized T-wave area x T-wave duration, corrected according to the Rautaharju formula, had a strong negative association with PRS (*β* = −0.14, *t*-Stat = −2.76, *p* = 0.006). Unlike the main effects that confirm a positive association with PRS, the interaction term of T-wave parameters suggests a negative relationship with PRS. Importantly, no significant effect of the 169 SNP-based PRS was observed for any of the other investigated parameters, either uncorrected or after heart-rate correction using the five methods (see Supplementary Table S1).

These findings suggest that the relationship between polygenic burden and ECG phenotype is modulated by temporal and morphological aspects of ventricular repolarization. Specifically, the negative interaction terms indicate that the influence of T-wave area and T-wave duration on PRS becomes attenuated at longer T-wave durations, consistent with a more complex interplay between repolarization timing and morphology in shaping the expression of genetic risk.

To assess whether nonlinear ECG–PRS relationships improved model performance, we repeated the analysis after expanding the predictor space to include quadratic terms for all ECG features. Although several quadratic terms were retained by elastic net selection, the resulting model showed reduced explanatory power (*R*^2^ = 0.07) compared with the parsimonious linear–interaction model (*R*^2^ = 0.09), indicating no overall benefit from higher-order terms.

## 4 Discussion

In this study, we investigated how a polygenic risk score (PRS) modulates T-wave geometry in a genetically homogeneous cohort of patients with congenital long QT syndrome type 1 (LQT1). Unlike previous investigations, our analysis focused on heart-rate corrected repolarization intervals and morphology features, applying five correction formulas to high-resolution digital ECG data. Using our recently validated algorithm for quantitative ECG analysis (24) and multivariate regression modeling, we identified two major findings.

Although the overall variance explained by the model was modest (*R*^2^ = 0.09), the relative contribution of individual predictors was not uniform. QTcB accounted for the strongest independent association with PRS (*β* = 0.24, *p* = 0.00008), expanding prior evidence that common genetic variation contributes to myocardial repolarization and QT prolongation (18, 31). In addition the T-peak to T-end (TPE) interval corrected according to Rautaharju (TPEcR) also showed a significant positive association with PRS (*β* = 0.16, *p* = 0.008), providing additional independent explanatory value beyond QTc and suggesting that polygenic background modulates not only global repolarization duration but also regional heterogeneity in repolarization timing.

In contrast, T-wave area and T-wave duration did not exhibit significant independent main effects, but we observed a novel association of PRS with a negative interaction between heart-rate corrected T-wave area and T-wave duration. This interaction contributed a smaller but statistically robust increment to the overall model, indicating that cumulative polygenic burden from the 169 investigated SNPs also affects the geometric and temporal properties of the T-wave—key determinants of ventricular repolarization. This finding aligns with the report by Ramirez et al. (33), which identified several SNPs with modifying effects on TPE interval duration under resting and stress conditions, further supporting the concept that common variants shape fine-grained electrophysiologic traits beyond overall QT prolongation.

Interestingly, none of the other digital ECG parameters, such as JT intervals or the R-to-T amplitude ratio, showed a significant relationship with PRS. Among the five correction methods applied for heart rate adjustment (Bazett, Fridericia, Framingham, Hodges, and Rautaharju), only the Bazett-corrected QTc, the Rautaharju-corrected TPE, and the Rautaharju-corrected T-wave geometry parameters (T-wave area and T-wave duration) emerged as significantly associated with PRS. This is important because the Rautaharju correction, unlike traditional formulas such as Bazett or Fridericia, applies a sex-specific linear model derived from large population datasets (30), offering superior accuracy across a wide range of RR intervals. The significant T-wave area x T-wave duration interaction, under this correction, suggests that the observed T-wave geometry<–>PRS relationships is probably not an artifact of heart rate variability but rather reflects intrinsic repolarization dynamics and characteristics. Moreover, Rautaharju correction may better capture subtle sex-specific modulation of ventricular repolarization, aligning with prior evidence that women exhibit longer QTc but lower dispersion of repolarization compared to men (10). Thus, the significant effect of the interaction between Rautaharju-corrected T-wave area and T-wave duration on PRS, strengthens the conclusion that polygenic burden modulates both temporal and morphological aspects of repolarization independently of heart rate. A comprehensive analysis of repolarization intervals beyond QT and JT—using high-resolution digital (raw) ECG data—has not previously been reported for patients with congenital LQTS.

The Bazett formula, despite its known rate-dependent influence on QTc calculation at low and high heart rates, still remains the most commonly used in both clinical and GWAS contexts. The lack of association with other correction formulas does not necessarily imply absence of any modulating effect, but rather indicates that population-derived PRS best capture the variance in QTc measured using standard conventions. This observation is consistent with the construction of the PRS applied here, which was derived from large population-based GWAS of QT interval duration and optimized for capturing common variant contributions to QT variability across the physiological spectrum (31). Future work using multi-trait PRS based on JT or TPE components may uncover additional associations.

The negative effect of the interaction between T-wave area and T-wave duration on PRS represents a novel insight into the morphological dimension of repolarization heterogeneity. A plausible physiological interpretation is that increased polygenic burden elongates the repolarization process while simultaneously flattening T-wave amplitude—an adaptive remodeling aimed at stabilizing repolarization gradients under increased electrical load. Prior studies have shown that broader, low-amplitude, or bifid T-waves are associated with increased arrhythmia susceptibility and more pronounced dispersion of repolarization (12, 13). Our findings extend this concept by suggesting that these morphological features may not only reflect monogenic ion channel dysfunction but also the additive influence of common genetic variants shaping ventricular recovery dynamics. In addition, these findings emphasize the complexity of genotype–phenotype relationships in congenital LQTS and underscore the importance of accounting for polygenic background when evaluating disease risk (16, 34, 35).

The significant association between TPEcR and PRS may reflect transmural dispersion of ventricular repolarization. TPE has been established as an independent predictor of arrhythmic risk across multiple clinical contexts. For instance, a large community-based case-control study, demonstrated that prolonged TPE on the resting ECG was independently associated with increased risk of sudden cardiac death in the general population, with mean TPE values significantly greater in sudden cardiac death cases (89.4 ms) compared to controls (79.5 ms) (9). This prognostic significance has been attributed to TPE's capacity to capture regional heterogeneity in repolarization, creating a substrate for reentrant arrhythmias. More recently, Ramírez et al. (33) conducted genome-wide association studies revealing that common genetic variants modulate the TPE interval, identifying 32 independent loci for resting TPE that influence ventricular repolarization as well as cardiac conduction and contraction. These findings establish TPE as a genetically determined trait influenced by polygenic architecture, consistent with our observation that PRS, capturing cumulative common variant effects, correlates with TPE duration in LQT1 patients. Furthermore, Takenaka et al. (36) demonstrated that exercise stress testing amplifies genotype-phenotype correlations in LQTS, with TPE increasing significantly during exercise in LQT1 but not in LQT2 patients, potentially explaining the higher incidence of exercise-triggered arrhythmic events in LQT1. Taken together, these studies support the biological plausibility of our finding that polygenic background modulates TPE in LQT1: common genetic variants not only extend overall repolarization (QTc) but also enhance transmural dispersion (TPE), thereby potentially amplifying arrhythmic vulnerability in individuals already predisposed by pathogenic KCNQ1 mutations. The fact that TPE showed significant association with PRS only when corrected using the Rautaharju formula, applying sex-specific linear correction, suggests that polygenic effects on transmural repolarization may be particularly sensitive to heart rate and sex-related modulation, warranting further investigation in larger cohorts with exercise testing protocols.

It is also noteworthy that the overall explanatory power of our regression model (*R*^2^ = 0.09) and the effect size of QTc (*β* = 0.24) and TPEcR (*β* = 0.16) on PRS were modest but statistically robust. This magnitude aligns with expectations for polygenic modulation within a monogenic context, where high-impact variants dominate disease causation but common variants subtly modify phenotypic expressivity and penetrance (5). While LQTS-specific PRSs have been developed to model disease severity and penetrance among carriers of pathogenic variants, such scores are optimized for case–control or modifier analyses rather than for continuous QT-related phenotypes. Importantly, these results demonstrate that integrating polygenic information may refine phenotype interpretation even within rare inherited diseases, supporting a more quantitative view of genetic architecture in LQT1.

The observation that only specific ECG features correlate with PRS highlights the trait specificity of polygenic influences on cardiac electrophysiology. PRS captures the additive contribution of common alleles tuned to population-level variance; thus, their effects may preferentially manifest in traits closely aligned with the discovery phenotype—here, QT interval duration. More specialized ECG indices, such as TPE or JT intervals, may require tailored PRS derived from dedicated GWAS of those traits. Methodologically, measurement noise, inter-lead variability, and the limited sample size may have further attenuated weaker associations. Nonetheless, the consistent directionality across models supports the biological plausibility of a polygenic–morphology link.

From a translational perspective, our findings emphasize that combining PRS with quantitative ECG analysis may enhance individualized risk assessment in inherited arrhythmia syndromes. Automated ECG analytics now enable reproducible extraction of subtle T-wave descriptors (24), while PRS offer scalable measures of genetic predisposition. Their integration could refine clinical decision-making, particularly in individuals with LQT1 and borderline QTc values or uncertain mutation pathogenicity. However, as effect sizes are small and the model explains a limited proportion of variance, PRS should currently be viewed as a complementary modifier rather than a standalone diagnostic tool.

Importantly, prior population-based studies have demonstrated that even minor ECG abnormalities—including subtle T-wave changes—are independently associated with an increased risk for future cardiac events, and their detection in otherwise healthy individuals can prompt early genetic evaluation or family screening (37, 38). Advances in automated ECG analysis now enable the identification and classification of nuanced T-wave morphology patterns that might previously have gone unnoticed, thereby improving the screening potential of standard ECGs (39). These technological advances, combined with polygenic risk modeling, suggest that quantitative ECG features could serve as accessible biomarkers bridging population genetics and clinical cardiology.

A further consideration is that genetic effects on cardiac repolarization are inherently nonlinear at the molecular and cellular levels. Functional studies in LQT1 have demonstrated that even single pathogenic KCNQ1 mutations exert heterogeneous and nonlinear effects on IKs current density and gating properties, resulting in variable electrophysiological and clinical expressivity [for instance in Jons et al. (40)]. Extending this concept, it is plausible that the cumulative effects of multiple common variants influencing repolarization may also manifest in nonlinear genotype–phenotype relationships, potentially limiting the performance of linear polygenic risk models. To address this possibility, we performed a sensitivity analysis explicitly incorporating quadratic ECG terms into the regression framework. Although several nonlinear terms were retained after regularization, this expanded model showed reduced explanatory power and substantially increased complexity compared with the parsimonious linear–interaction model, indicating that higher-order terms did not meaningfully improve the relationship between ECG morphology and aggregate polygenic burden in this cohort. These findings suggest that, while nonlinearity is fundamental at the ion-channel level, such effects are not readily captured by conventional PRS frameworks derived from marginal population-level associations. Notably, the SNPs included in the PRS are predominantly noncoding GWAS variants for which mutation-specific functional effects on IKs are not available, precluding function-based differential weighting analogous to monogenic LQT1 mutations. Future approaches that integrate functional genomics, pathway-aware weighting, or biophysical modeling may therefore be required to better represent nonlinear genotype–phenotype relationships underlying ventricular repolarization.

## 5 Future implications

Beyond its relevance for LQT1, our methodological framework provides a scalable blueprint for integrating high-resolution ECG phenotyping with polygenic risk analysis in more prevalent cardiovascular disorders. The combination of automated signal extraction, robust feature engineering, and standardized PRS computation can be readily adapted to conditions such as drug-induced QT prolongation (15, 16), cardiomyopathies, or atrial fibrillation, where subtle electrical changes precede overt disease manifestation. This scalability underscores the potential of our approach as a model for future genotype–phenotype studies in precision cardiology. In parallel, the integration of artificial intelligence–driven ECG analytics with polygenic modeling may further enhance the detection of complex genotype–phenotype interactions and enable real-time, personalized risk prediction in inherited and acquired arrhythmia syndromes.

The integration of polygenic risk scoring into clinical workflows holds considerable promise for enhancing risk stratification, particularly among patients with ambiguous or incomplete genetic findings. Quantifying polygenic burden could facilitate earlier clinical interventions, enable personalized monitoring strategies, and support targeted screening of at-risk family members (41). The advent of automated ECG analysis algorithms, as demonstrated in the present study, further amplifies this potential by enabling reproducible, high-throughput extraction of quantitative repolarization metrics that complement PRS-based risk assessment. To realize this potential, prospective studies are required to validate PRS-guided risk models, explore the integration of artificial intelligence and machine learning approaches for enhanced phenotype recognition, and assess their combined impact on clinical outcomes. Collectively, these observations underscore the growing value of integrating genomic information with AI-augmented, quantitative ECG analytics to detect early repolarization abnormalities and optimize arrhythmic risk management in inherited channelopathies (35).

## 6 Limitations

Our findings should be interpreted in the context of several limitations. First, the study cohort consisted exclusively of European patients with LQT1, which may limit the generalizability of the results to other ancestral populations. In addition, the cross-sectional design precludes causal inference, and arrhythmic outcomes were not analyzed, preventing assessment of the clinical implications of the observed ECG–PRS associations. The polygenic risk score used in this study was derived from population-based QTc GWAS and was not specifically optimized to capture modifier effects within LQTS; therefore, disease-specific PRSs may account for additional variance related to penetrance or clinical severity. Prospective, multi-ancestry studies incorporating longitudinal ECG monitoring and LQTS-specific PRSs are needed to validate and extend these findings.

Another important consideration relates to the physiological context in which ECG measurements were obtained. Because LQT1-related arrhythmic events are typically triggered under *β*-adrenergic stimulation, it remains unclear whether the observed PRS–ECG associations at rest persist, attenuate, or amplify during sympathetic activation. It is biologically plausible that polygenic background influencing baseline repolarization phenotypes may also modulate dynamic QT adaptation and T-wave morphology under adrenergic stress. However, exercise or pharmacologic stress testing was not available in the present cohort, and prospective studies are required to address this question.

## 7 Conclusions

In summary, our study demonstrates that polygenic background modulates both the duration and morphology of ventricular repolarization in LQT1. The significant association between PRS and QTcB, together with the novel Rautaharju-corrected TPEcR main effect and T-wave area × T-wave duration interaction, underscores the importance of considering common genetic variation when interpreting ECG phenotypes in monogenic arrhythmia syndromes. These results further lay the groundwork for integrating PRS-informed digital ECG analytics into precision cardiology frameworks for inherited disorders of cardiac repolarization as a hypothesis-generating and phenotypic stratification tool.

## Data Availability

The original contributions presented in the study are publicly available. This data can be found here: https://doi.org/10.17879/5hhhq-r2s22.
