# Supplementary material for: Polygenic risk modulates myocardial repolarization and T-wave geometry in congenital long-QT syndrome type 1: evidence from digital ECG phenotyping
Source: Front Cardiovasc Med. 2026 Apr 23;13:1736409. doi: 10.3389/fcvm.2026.1736409 (PMC13149143; doi:10.3389/fcvm.2026.1736409)
Supplement: Supplementary file 1 [file Datasheet1.pdf]

Supplementary Materials

Table 1: Calculated ECG parameters, corrected according to five different methods

	ECG Parameters (ms)	Mean Male	Std Male	Mean Female	Std Female
Corrected according to Bazett	TPE	70.7450	12.0220	71.7912	14.8111
	TPE / QT ratio	0.1696	0.0350	0.1643	0.0356
	T-wave area (TWA)	16.1610	13.1878	14.8679	11.7795
	T-wave duration (TWD)	279.3161	74.6474	249.7550	71.8710
	JT-start	88.5032	62.8945	126.1369	65.1667
	JT-peak	289.3210	34.9169	299.4686	32.9153
	JT-end	364.6365	39.0292	375.6932	38.2523
	T-start to T-peak	203.3853	61.4355	177.7905	59.7726
Corrected according to Fridericia	TPE	69.9304	12.0943	71.1489	14.4998
	TPE / QT ratio	0.1668	0.0293	0.1623	0.0319
	T-wave area (TWA)	15.9835	13.0607	14.7819	11.6852
	T-wave duration (TWD)	276.3140	75.0111	247.3705	70.2372
	JT-start	87.2188	61.4847	125.4609	65.1165
	JT-peak	286.0385	35.8042	297.1804	34.8212
	JT-end	360.3698	39.4592	372.6901	39.6745
	T-start to T-peak	201.3084	61.6548	176.1063	58.5641
Corrected according to Hodges	TPE	68.5846	14.0734	70.0153	15.1333
	TPE / QT ratio	0.0572	0.0242	0.0541	0.0268
	T-wave area (TWA)	15.6315	13.0943	14.5618	11.6741
	T-wave duration (TWD)	271.6752	80.3758	243.3785	70.1377
	JT-start	85.0610	59.9285	124.4478	66.4036
	JT-peak	280.8984	45.9491	293.5506	45.3534
	JT-end	353.6882	52.6423	367.9023	52.1676

	T-start to T-peak	198.0886	65.0443	173.2555	58.1968
Corrected according to Framingham	TPE	68.6958	14.0541	70.1249	15.1235
	TPE / QT ratio	0.1685	0.0421	0.1635	0.0442
	T-wave area (TWA)	15.7329	13.0916	14.6686	11.6633
	T-wave duration (TWD)	271.7865	80.3609	243.4881	70.1329
	JT-start	85.1723	59.9265	124.5574	66.3932
	JT-peak	281.0097	45.9257	293.6602	45.3321
	JT-end	353.7994	52.6181	368.0119	52.1468
	T-start to T-peak	198.1999	65.0300	173.3651	58.1926
Corrected according to Rautaharju	TPE	68.7013	14.0374	70.1289	15.1151
	TPE / QT ratio	0.1746	0.0679	0.1679	0.0661
	T-wave area (TWA)	15.7357	13.0873	14.6722	11.6569
	T-wave duration (TWD)	271.7919	80.3479	243.4921	70.1286
	JT-start	85.1778	59.9247	124.5614	66.3841
	JT-peak	281.0151	45.9053	293.6642	45.3137
	JT-end	353.8049	52.5972	368.0159	52.1287
	T-start to T-peak	198.2053	65.0175	173.3691	58.1890

Table 2: Summary of Single Nucleotide Polymorphisms (SNPs) Included in the Polygenic Risk Score (PRS)

rsID Lead variant	Chromsomal position	Nearest transcript	Chr.	Position	Effect allele (EA)	Reported frequency EA	Beta (ms)
rs706007	1:10780727:A:G	*CASZ1*	1	10780727	A	0,8518	-0,66
rs11102093	1:110901129:A:G	*SLC16A4*	1	110901129	A	0,9125	-0,63
rs6670224	1:113434105:A:G	*RP3-522D1.2*	1	113434105	A	0,4194	0,36
rs35951215	1:116280144:A:G	*CASQ2*	1	116280144	A	0,3354	-0,31
rs75056606	1:150718602:G:T	*CTSS*	1	150718602	T	0,8566	-0,51
rs4364874	1:154167788:A:C	*TPM3*	1	154167788	A	0,5697	-0,37
rs12143842	1:162033890:C:T	*NOS1AP*	1	162033890	T	0,245	3,68
rs1591734	1:169271902:C:T	*DPT/ATP1B1*	1	169271902	T	0,1432	-1,80
rs577676	1:170587340:C:T	*RP1-79C4.1*	1	170587340	T	0,4724	-0,41
rs4951258	1:205691316:A:G	*NUCKS1*	1	205691316	A	0,3778	-0,51
rs11119840	1:212246168:C:T	*DTL*	1	212246168	T	0,4725	-0,35
rs77510181	1:214268170:C:T	*PROX1*	1	214268170	T	0,9202	0,49
rs2503715	1:2144107:A:G	*C1orf86*	1	2144107	A	0,1291	0,91
rs73096374	1:227445096:A:G	*CDC42BPA*	1	227445096	A	0,1387	0,42
rs2298632	1:23710475:C:T	*TCEA3*	1	23710475	T	0,4819	0,76
rs116015634	1:41250961:C:T	*KCNQ4*	1	41250961	T	0,0196	-3,19
rs1997997	1:61886046:A:G	*NFIA*	1	61886046	A	0,5083	0,41
rs846111	1:6279370:C:G	*RNF207*	1	6279370	C	0,2627	1,83
rs72677052	1:66984572:A:G	*SGIP1*	1	66984572	A	0,2213	0,67
rs1166708	1:78331226:C:T	*FAM73A*	1	78331226	T	0,2063	0,44
rs12127491	1:9140327:C:T	*SLC2A5*	1	9140327	T	0,2182	-0,37
rs72845663	10:104081349:C:T	*GBF1*	10	104081349	T	0,3771	-0,48
rs10886369	10:120783641:A:G	*NANOS1*	10	120783641	A	0,6107	-0,29
rs146042014	10:18629941:A:G	*CACNB2*	10	18629941	A	0,007	2,81
rs11013167	10:23163178:A:G	*ARMC3*	10	23163178	A	0,7452	0,37
rs176833	10:38365283:A:G	*ZNF37A*	10	38365283	A	0,4838	-0,41
rs4948721	10:43854771:C:G	*FXYD4*	10	43854771	C	0,3747	0,33
rs55780106	10:50531606:A:C	*C10orf71*	10	50531606	A	0,1717	0,43
rs4746140	10:75417249:C:G	*SYNPO2L*	10	75417249	C	0,2119	0,40
rs1658323	10:80874523:C:T	*ZMIZ1*	10	80874523	T	0,366	-0,35
rs117524646	10:91278750:A:T	*SLC16A12*	10	91278750	A	0,9553	-0,85
rs10748555	10:92682060:A:C	*RNU6-740P*	10	92682060	A	0,4882	0,36
rs307211	11:13521274:G:T	*PTH*	11	13521274	T	0,174	0,47

rs985136	11:17497794:C:G	*ABCC8*	11	17497794	C	0,5009	0,45
rs2074238	11:2484803:C:T	*KCNQ1*	11	2484803	T	0,0875	-4,74
rs61884689	11:30453901:G:T	*MPPED2*	11	30453901	T	0,1453	-0,52
rs7395231	11:48091689:C:T	*MYBPC3*	11	48091689	T	0,3365	0,32
rs174537	11:61552680:G:T	*FEN1-FADS2/ TMEM258*	11	61552680	T	0,342	-0,61
rs1346	11:65337251:A:T	*SSSCA1-AS1*	11	65337251	A	0,8092	-0,44
rs12315174	12:110586278:A:G	*ATP2A2*	12	110586278	A	0,2273	0,95
rs7977083	12:114790500:A:G	*TBX5*	12	114790500	A	0,2752	0,39
rs76382809	12:120671647:C:T	*PXN*	12	120671647	T	0,0238	-1,45
rs11048525	12:26598990:A:C	*ITPR2*	12	26598990	A	0,9727	-1,23
rs2045172	12:32980161:G:T	*PKP2B*	12	32980161	T	0,1446	0,58
rs78341918	12:46199798:C:T	*ARID2*	12	46199798	T	0,9666	1,51
rs10849144	12:4899032:C:T	*GALNT8*	12	4899032	T	0,4863	0,30
rs9549705	13:113888425:C:T	*CUL4A*	13	113888425	T	0,7673	0,46
rs1747221	13:47225573:G:T	*LRCH1*	13	47225573	T	0,6781	-0,46
rs17061696	13:74511991:C:G	*KLF12*	13	74511991	C	0,3671	0,78
rs2273905	14:102974999:C:T	*ANKRD9*	14	102974999	T	0,3434	0,54
rs17782683	14:75167706:A:G	*AREL1:AC0079 56.1*	14	75167706	A	0,498	-0,35
rs57882498	14:90538964:A:G	*KCNK13*	14	90538964	A	0,2014	0,41
rs3743038	15:41694743:C:T	*NDUFAF1*	15	41694743	T	0,3304	0,35
rs3098202	15:50838236:C:G	*USP50-TRPM7*	15	50838236	C	0,5393	-0,63
rs12441088	15:78928264:G:T	*CHRNB4*	15	78928264	T	0,7238	-0,36
rs2115630	15:85364516:C:T	*ZNF592*	15	85364516	T	0,4623	0,49
rs7191330	16:11692658:C:T	*LITAF*	16	11692658	T	0,4561	-1,33
rs30226	16:14405892:A:G	*MIR193BHG/ MKL2*	16	14405892	A	0,4532	0,67
rs45492694	16:16156634:A:C	*ABCC1*	16	16156634	A	0,9038	-0,71
rs12448482	16:28838073:A:G	*ATP2A1*	16	28838073	A	0,3321	0,93
rs1296720	16:3873642:A:C	*CREBBP*	16	3873642	A	0,807	-0,72
rs1186768	16:54618689:A:G	*AC079412.1*	16	54618689	A	0,1594	0,43
rs4485352	16:58601410:A:G	*CNOT1/SETD6/ NDRG4/GINS3*	16	58601410	A	0,2603	-1,93
rs59985111	16:615326:C:T	*C16orf11*	16	615326	T	0,5504	-0,43
rs186400483	16:73508330:A:G	*RP11-44L9.1*	16	73508330	A	0,0236	-1,04
rs4614769	17:12155901:A:G	*RP11-471L13.2*	17	12155901	A	0,4874	-0,34
rs1804193	17:15134006:C:T	*PMP22*	17	15134006	T	0,0773	0,62
rs9904351	17:17624193:C:T	*RAI1*	17	17624193	T	0,3452	0,38

rs8072677	17:25614043:G:T	*RP11-173M1.3*	17	25614043	T	0,3819	-0,33
rs11078312	17:2600186:A:G	*CLUH*	17	2600186	A	0,1213	0,63
rs652677	17:26904517:A:C	*CRYBA1*	17	26904517	A	0,2356	-0,40
rs10512436	17:30062860:C:G	*RNU6-1134P*	17	30062860	C	0,0766	-0,68
rs1052536	17:33331575:C:T	*LIG3*	17	33331575	T	0,4459	-0,83
rs3764351	17:37824339:A:G	*PNMT*	17	37824339	A	0,6296	-0,30
rs17608766	17:45013271:C:T	*GOSR2*	17	45013271	T	0,8638	-0,77
rs3803886	17:49257790:C:T	*MBTD1*	17	49257790	T	0,5263	-0,34
rs9915847	17:57473166:A:G	*YPEL2*	17	57473166	A	0,4945	0,70
rs9890911	17:64315569:C:T	*PRKCA*	17	64315569	T	0,4244	-0,86
rs12941598	17:68453327:A:G	*KCNJ2*	17	68453327	A	0,2176	-1,24
rs3809811	17:7224319:C:T	*NEURL4*	17	7224319	T	0,4731	-0,36
rs150419024	18:24170539:A:G	*KCTD1*	18	24170539	A	0,9864	-1,54
rs145439349	19:11292839:A:G	*KANK2*	19	11292839	A	0,2695	-0,36
rs463200	19:22327489:C:T	*RP11-157B13.3*	19	22327489	T	0,6185	0,32
rs11085015	19:3369572:G:T	*NFIC*	19	3369572	T	0,1966	-0,52
rs8106249	19:46862470:A:T	*PPP5C*	19	46862470	A	0,6836	0,46
rs11666224	19:49571399:G:T	*KCNA7*	19	49571399	T	0,8799	0,53
rs73068914	19:56075451:C:T	*CTD-2537I9.5*	19	56075451	T	0,3199	-0,41
rs661821	19:7582649:C:T	*CTD-2207O23.12:ZNF358*	19	7582649	T	0,3737	-0,59
rs2261682	2:128620372:C:T	*AMMECR1L*	2	128620372	T	0,506	-0,33
rs3754781	2:169015476:A:G	*STK39*	2	169015476	A	0,1643	0,38
rs35717017	2:174888351:G:T	*SP3*	2	174888351	T	0,1978	0,84
rs66677602	2:179611711:A:C	*TTN-CCDC141*	2	179611711	A	0,0633	1,38
rs72914308	2:192750798:A:G	*AC098617.1*	2	192750798	A	0,0961	0,69
rs4673904	2:201163454:A:T	*SPATS2L*	2	201163454	A	0,6161	-0,45
rs35394392	2:220500830:C:G	*SLC4A3*	2	220500830	C	0,2133	0,82
rs2971870	2:234379522:C:G	*DGKD*	2	234379522	C	0,1987	-0,37
rs1275983	2:26910556:C:T	*KCNK3*	2	26910556	T	0,357	0,38
rs10490255	2:40738240:C:T	*SLC8A1*	2	40738240	T	0,9122	1,73
rs7608165	2:74202473:C:T	*DGUOK-AS1*	2	74202473	T	0,6724	0,36
rs186606513	2:97482001:A:G	*CNNM3*	2	97482001	A	0,0229	-1,28
rs7273470	20:33456921:C:G	*GGT7*	20	33456921	C	0,2374	-0,44
rs2427312	20:60970591:C:T	*CABLES2*	20	60970591	T	0,1886	-0,50
rs2832274	21:30600189:A:T	*LINC00189*	21	30600189	A	0,6191	-0,43
rs1805128	21:35821680:C:T	*KCNE1*	21	35821680	T	0,0139	7,13
rs2836950	21:40604429:C:G	*BRWD1*	21	40604429	C	0,6435	-0,37

rs196067	22:38853677:C:T	*KCNJ4*	22	38853677	T	0,6371	0,55
rs4688023	3:119379186:C:T	*POPDC2:COX17*	3	119379186	T	0,4455	0,29
rs73186030	3:122013465:C:T	*CASR*	3	122013465	T	0,1301	-0,83
rs11721024	3:141602770:G:T	*ATP1B3*	3	141602770	T	0,148	0,47
rs7430029	3:149986210:C:T	*RP11-483E7.1*	3	149986210	T	0,8622	-0,54
rs3087964	3:185348402:C:T	*SENP2*	3	185348402	T	0,3431	0,42
rs1706003	3:194299967:G:T	*TMEM44*	3	194299967	T	0,4378	-0,73
rs476778	3:21975294:A:C	*ZNF385D*	3	21975294	A	0,8358	0,42
rs73032363	3:24472866:A:G	*THRB*	3	24472866	A	0,7124	-0,41
rs7373492	3:38687369:C:T	*SCN5A-SCN10A*	3	38687369	T	0,0296	3,93
rs11921457	3:42103045:G:T	*TRAK1*	3	42103045	T	0,8205	0,44
rs12487736	3:47459679:C:T	*C3ORF75*	3	47459679	T	0,5502	0,36
rs7373072	3:48960017:A:T	*ARIH2*	3	48960017	A	0,7445	0,41
rs62251027	3:69862454:C:G	*MITF*	3	69862454	C	0,7971	0,50
rs4013	4:103552813:C:T	*NFKB1*	4	103552813	T	0,532	0,30
rs4834347	4:114425934:A:C	*CAMK2D*	4	114425934	A	0,2405	0,38
rs6820265	4:141903402:C:T	*RNF150*	4	141903402	T	0,309	0,42
rs1560941	4:154378744:A:G	*KIAA0922*	4	154378744	A	0,618	0,39
rs78243103	4:174581749:A:G	*RANP6*	4	174581749	A	0,0482	1,04
rs11734408	4:23882519:A:G	*PPARGC1A*	4	23882519	A	0,7189	-0,41
rs72627509	4:57839051:C:G	*NOA1*	4	57839051	C	0,8155	0,37
rs34411702	4:6835635:A:G	*KIAA0232*	4	6835635	A	0,1447	-0,47
rs1947378	4:72138073:A:T	*SLC4A4*	4	72138073	A	0,8867	-1,06
rs10009824	4:83926921:A:C	*LIN54*	4	83926921	A	0,4522	-0,48
rs10028613	4:95223698:C:T	*SMARCAD1*	4	95223698	T	0,3914	0,43
rs1811677	5:10456336:C:T	*ROPN1L*	5	10456336	T	0,7714	0,50
rs56078134	5:1065683:A:C	*SLC12A7*	5	1065683	A	0,492	-0,57
rs329124	5:133865452:A:G	*JADE2*	5	133865452	A	0,5618	-0,34
rs13355516	5:137380603:A:G	*WNT8A/GFRA3*	5	137380603	A	0,8324	1,00
rs7733550	5:153906061:C:T	*CTB-158E9.2*	5	153906061	T	0,7214	0,35
rs55893552	5:172650620:A:T	*NKX2-5*	5	172650620	A	0,1795	0,77
rs145660890	5:178968434:A:G	*RUFY1*	5	178968434	A	0,0086	2,83
rs7737409	5:56994529:A:C	*RP11-772C9.1*	5	56994529	A	0,2082	-0,69
rs255390	5:61806707:A:G	*KIF2A:IPO11*	5	61806707	A	0,3435	-0,30
rs17774009	6:105545057:A:G	*BVES*	6	105545057	A	0,0634	1,24

rs11153730	6:118667522:C:T	*SLC35F1/ C6orf204/ CEP85L*	6	118667522	T	0,5337	-1,73
rs1977141	6:126097457:A:G	*NCOA7*	6	126097457	A	0,4045	0,39
rs2627230	6:134379869:A:T	*SLC2A12*	6	134379869	A	0,5871	0,43
rs6914805	6:16281187:C:T	*GMPR*	6	16281187	T	0,2913	0,51
rs206705	6:164528867:A:T	*RP1-155D22.1*	6	164528867	A	0,3039	-0,45
rs34969716	6:18210109:A:G	*KDM1B*	6	18210109	A	0,2925	-0,78
rs394657	6:32187023:A:G	*HLA-DRB5*	6	32187023	A	0,5618	0,36
rs3176326	6:36647289:A:G	*CDKN1A*	6	36647289	A	0,2031	-0,38
rs9379066	6:6901856:C:T	*RP3-429O6.1*	6	6901856	T	0,6426	0,40
rs2897358	7:100088640:C:G	*NYAP1*	7	100088640	C	0,8033	-0,54
rs7467	7:112116198:A:G	*IFRD1*	7	112116198	A	0,3139	0,34
rs1997571	7:116198621:A:G	*CAV1*	7	116198621	A	0,5771	-0,56
rs758890	7:150655643:A:G	*KCNH2*	7	150655643	A	0,3536	1,57
rs340395	7:35411626:C:G	*AC009531.2*	7	35411626	C	0,3976	-0,28
rs17538328	7:40134678:A:G	*CDK13*	7	40134678	A	0,8658	0,71
rs3211938	7:80300449:G:T	*CD36*	7	80300449	T	0,9304	-2,13
rs2158806	7:95051998:A:C	*PON2*	7	95051998	A	0,7581	0,41
rs599777	8:103931938:C:G	*AZIN1*	8	103931938	C	0,4207	0,46
rs72671655	8:106347897:A:T	*ZFPM2*	8	106347897	A	0,0381	1,24
rs67839940	8:133845436:A:G	*PHF20L1*	8	133845436	A	0,7813	0,39
rs17060733	8:21866933:C:T	*XPO7*	8	21866933	T	0,8236	-0,54
rs74783055	8:71277238:C:T	*NCOA2*	8	71277238	T	0,8956	-0,96
rs1317572	8:98804691:C:T	*LAPTM4B*	8	98804691	T	0,3909	-0,64
rs2489363	9:103343155:G:T	*MURC*	9	103343155	T	0,4347	-0,30
rs74552897	9:109459872:A:G	*RP11-308N19.4*	9	109459872	A	0,0215	1,30
rs4740240	9:134320354:G:T	*PRRC2B*	9	134320354	T	0,0562	0,65
rs78078655	9:34994171:C:T	*DNAJB5*	9	34994171	T	0,0076	2,50
rs6477028	9:6024844:G:T	*RANBP6*	9	6024844	T	0,254	-0,30
rs356131	9:97567327:A:G	*C9orf3*	9	97567327	A	0,409	0,44
